# CHRONIC PAIN, PAIN INTERFERENCES, AND COGNITION AMONG MIDDLE- AND OLD-AGED AMERICANS: A FIXED-EFFECTS ANALYSIS

**DOI:** 10.1093/geroni/igae098.2649

**Published:** 2024-12-31

**Authors:** Chang Yu, Ashley Barr, Yulin Yang, Hanna Grol-Prokopczyk

**Affiliations:** University at Buffalo, SUNY, Buffalo, New York, United States; University at Buffalo, SUNY, Buffalo, New York, United States; University of California San Francisco, San Francisco, California, United States; University at Buffalo, SUNY, Buffalo, New York, United States

## Abstract

**Objectives:**

The current study examines the relationship between chronic pain, chronic pain interference, and cognitive function in middle-aged and older Americans, focusing on how these associations may vary by educational background. Method: Data were obtained from the 2004-2006 and 2013-2017 waves of the Midlife in the United States Study (MIDUS 2 and MIDUS 3, N=6266 observations nested with 3965 individuals). We employed fixed-effects linear models to examine within-individual changes, specifically, the relationship between changes in chronic pain, pain interferences (with activity, mood, relations, sleep, and enjoyment) and their impact on cognitive function (specifically episodic memory and executive function). In addition, group differences in these fixed effects by education were examined via interaction terms.

**Results:**

The results indicated that changes in pain negatively correlated with episodic memory, but this association was observed only for individuals without any college. Additionally, changes in pain interferences showed a negative correlation with both episodic memory and executive functioning across all levels of educational attainment.

**Discussion:**

Findings highlight the importance of not only considering the role of persistent pain but also the effects of pain interferences on cognitive decline among aging population.

